# Comprehensive Analysis of Major Latex-Like Protein Family Genes in Cucumber (*Cucumis sativus* L.) and Their Potential Roles in Phytophthora Blight Resistance

**DOI:** 10.3390/ijms24010784

**Published:** 2023-01-02

**Authors:** Yunyan Kang, Jiale Tong, Wei Liu, Zhongli Jiang, Gengzheng Pan, Xianpeng Ning, Xian Yang, Min Zhong

**Affiliations:** Department of Vegetable Science, College of Horticulture, South China Agricultural University, Guangzhou 510642, China

**Keywords:** genome-wide, major latex-like protein, expression analysis, *Phytophthora melonis*, *Cucumis sativus*

## Abstract

Major latex-like proteins (MLPs) play crucial roles in abiotic and biotic stresses. However, little was known about this gene family in cucumbers. In this study, a total of 37 putative cucumber *MLP* genes were identified on a genome-wide level and classified into three groups by sequence homologous comparison with *Arabidopsis thaliana*. Chromosome mapping suggested that only tandem duplication occurred in evolution. The multiple regulatory *cis*-elements related to stress, hormone, light and growth response were found in the promoter region of these *CsMLP* genes, indicating that *CsMLPs* might be widely involved in the process of plant growth, development and various stress conditions. Transcriptome analysis indicated a strong reprogramming of *MLPs* expression in response to *Phytophthora melonis* infection in cucumber. Knockdown of *CsMLP1* reduced the *P. melonis* tolerance, while transient overexpression of *CsMLP1* improved disease tolerance in cucumber. Conversely, the silence of *CsMLP5* decreased the lesion area caused by *P. melonis* in the cotyledons, and overexpression of *CsMLP5* promoted lesion expansion. Taken together, our results provide a comprehensive basis for further mining the function of *CsMLP* members and will also be significant for elucidating the evolutionary relationship in cucumber.

## 1. Introduction

Phytophthora fruit rot caused by *Phytophthora* spp. is a devastating soil-borne disease worldwide and has resulted in severe yield and quality losses in around 20 families of vegetable crops. In particular, in most susceptible Solanaceae and Cucurbitaceae hosts, *P. capsici* may cause foliar blighting, damping-off, wilting, and root, stem, and fruit rot. Losses of approximately 90% of processing squash in Michigan and 50% of hydroponically grown cucumbers in Mexico were reported due to *P. capsici* infection [1,2]. Phytophthora blight in field- and greenhouse-grown cucumber, squash, pepper and tomato were also observed in Canada. Losses of 10% plant population in a greenhouse pepper in 2006–2007; 5%, 5%, and 10% yield loss in commercial greenhouse tomatoes in 2007, 2008, and 2011, respectively, were observed in Leamington, Ontario [3]. In both the Guangxi Zhuang Autonomous Region and the Guangdong province, the two largest wax gourd and cucumber production regions in China, at least 10% losses in commercial cucurbit crops caused by *P. melonis* is observed annually, and the yield loss could reach up to 80% in the extremely rainy season [4]. Until now, the execution of aggressive integrated management measures still shows weak disease control when climate conditions are favorable for disease epidemics. Hence, exploiting disease-resistance genes and successfully breeding commercially-viable resistant varieties are effective solutions to manage phytophthora blight and prevent economic losses caused by *Phytophthora* spp. infection.

Major latex-like proteins (MLPs) belong to the second largest family of the Bet v 1 superfamily and are homologous to pathogenesis-related proteins class 10 (PR-10s) and Bet v 1s. Bet v_1 protein is characterized by the hydrophobic pocket binding various hydrophobic compounds and a rigid and highly conserved glycine-rich loop (GlyxGlyGlyxGly(Thr/Ser) [5,6]. PR-10s are found to exert RNase activity through the binding of their glycine-rich loop to bacteria and fungi RNA [7,8]. Although MLPs include a glycine-rich loop, MLPs lacking RNase activity have been demonstrated and also have been found to exhibit indirect resistance against pathogen infections via triggering *PR* genes [6]. For instance, MLP-PG1 did not show RNase activity in zucchini, and overexpression of this gene in tobacco plants produced *Botrytis cinerea* resistance, which was accompanied by substantially up-regulated two PR genes *PR*-2 and *PR-5* [9]. Nevertheless, it has also been documented that different MLPs induced resistance through their own distinct roles. For example, overexpression and T-DNA mutants demonstrated the positive contribution of *BvMLP1* and *BvMLP3* to *Rhizoctonia solani* resistance in sugar beet, whereas addictive influences were observed in *Atmlp1*/*Atmlp3* double mutants [10]. In cotton plants, *GhMLP28* was found to interact with cotton ethylene response factor 6 (GhERF6) and facilitated GhERF6 specifically binding to GCC box, consequently protecting cotton plants against *Verticillium dahliae* infection [11]. In addition, mulberry *MLPL329* conferred resistance against *Botrytis cinerea*, *Pseudomonas syringae* pv tomato DC3000 and phytoplasma [12], and *Nicotiana benthamiana* MLP28 operated *Potato virus Y* resistance [13]. He et al. [14] reported that overexpressing *MdMLP423* decreased apple resistance to *Botryosphaeria berengeriana* f. sp. *piricola* and *Alternaria alternata* apple pathotype (AAAP) infections by inhibiting the expression of genes related to defense- and stress-related genes and transcription factors. These results imply that MLPs might play an important role in pathogen defense in higher plants.

In cucurbit vegetables, one important function of MLP proteins was responsible for the uptake of persistent organic pollutants [15]. Moreover, in *Cucumis melo*, MLP proteins were identified in the stem phloem sap of cucumber mosaic virus-infected plants [16]. In *Cucumis sativus*, phloem MLP-like protein 328 was suggested as a potential signaling component in mediating brassinosteroid-regulated systemic resistance [17]. And MLP-like protein 328-like, MLP-like protein 329-like and MLP-like protein 423-like in the stem phloem [18], and MLP-like protein 328-like in the roots [19] were greatly associated with cucumber salinity stress tolerance. Qi et al. [20] also found that cucumber MLP-like protein 328 was significantly induced in response to aphid infestation. Collectively, these findings strongly support that MLP proteins in Cucurbitaceae vegetables are extensively involved in triggering a wide range of biotic and abiotic stress responses. However, until now, in cucurbit vegetables, *MLP* genes were only identified in *Cucurbita pepo* at the genomic level [21]. Genome-wide identification of *MLP* genes from cucumber and their function in disease resistance remains largely unclear.

In this study, we totally identified 37 MLP genes from the cucumber genome and comprehensively analyzed gene structure, conserved motifs, chromosomal localization, and *cis*-element. RNA-Seq analysis revealed MLP genes potentially connected to Phytophthora blight resistance, wherein two *MLP* genes, *CsMLP1* (CsaV3_1G045200) and *CsMLP5* (CsaV3_3G019100), showed higher transcriptional activity in the infected hypocotyl of resistant wild-type (WT) genotype ‘CCMC’ compared to susceptible genotype *cyp85a1* (EMS-mutated brassinosteroid-deficient mutant) on 72 h after *P. melonis* inoculation. Furthermore, overexpressing *CsMLP1* in WT cucumber plants enhanced resistance against *P. melonis* infection, but overexpression of *CsMLP5* reduced *P. melonis* resistance. Silencing of *CsMLP1* and *CsMLP5* further validated that *CsMLP1* positively and *CsMLP5* negatively regulated defense against *P. melonis* infection, respectively. Conclusively, this study serves as the first characterization of *CsMLP* genes at the whole genome level and their function in response to *P. melonis* infection, and these results will facilitate the breeding of disease-resistance Cucurbitaceae varieties through constructing new candidate genes of the MLP subfamily.

## 2. Results

### 2.1. Genome-Wide Identification of CsMLP Genes in Cucumber

**Figure 1:**
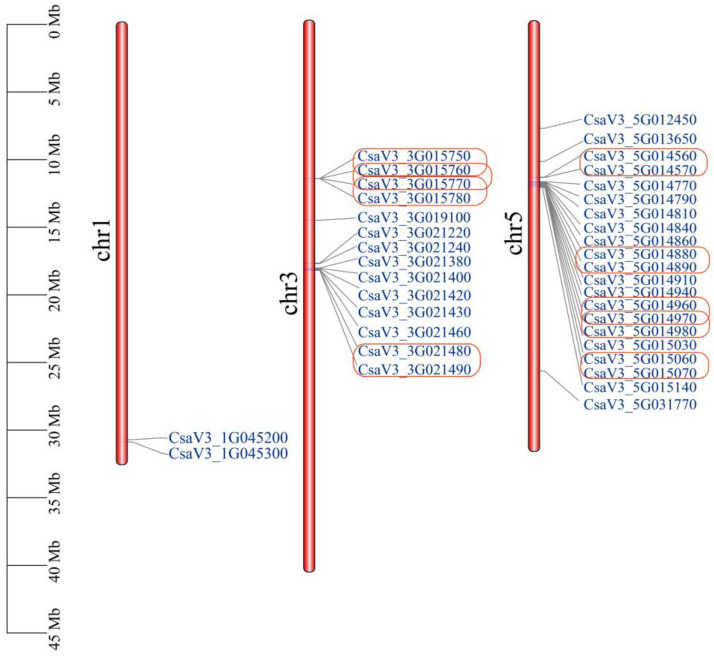
Chromosomal map and duplication events of *CsMLP* genes. Tandem duplication was indicated by red boxes.

A total of 37 putative sequences of *CsMLP* genes with complete Bet v 1 allergen domain was identified in the genome of *Cucumis sativus*. Then, we numbered the genes CsMLP1 to CsMLP37 based on their chromosomal locations (Table 1, Figure 1 and Figure S2). The length of CsMLP proteins ranged from 151 (CsMLP25) to 191 (CsMLP10) amino acids, and the predicted molecular weights ranged from 17.031 kDa (CsMLP6) to 21.7489 kDa (CsMLP10). The predicted pI-values of all CsMLP proteins were between 4.53 (CsMLP6) and 9.21 (CsMLP9; Table 1).

### 2.2. Chromosomal Location and Gene Duplication

Chromosomal distribution showed that 37 *CsMLPs* were unevenly distributed on three chromosomes. Among them, two genes were located on chromosome 1, 14 genes on chromosome 3, and 21 genes on chromosome 5 (Table 1, Figure 1).

We further analyzed the gene duplication of *CsMLP* genes in cucumber. As shown in Figure 1, four tandem duplication event (within the red box) was identified on chromosome 3 and five tandem duplication event in chromosome 5. The chromosome location of nine pairs of *CsMLP* genes was close in distance and was inserted by less than one gene. No segmental duplication events were detected (Figure 1). The results implied that tandem duplication events made contributions to the expansion of the cucumber *CsMLP* gene family.

### 2.3. Phylogenetic Tree of CsMLPs

**Figure 2:**
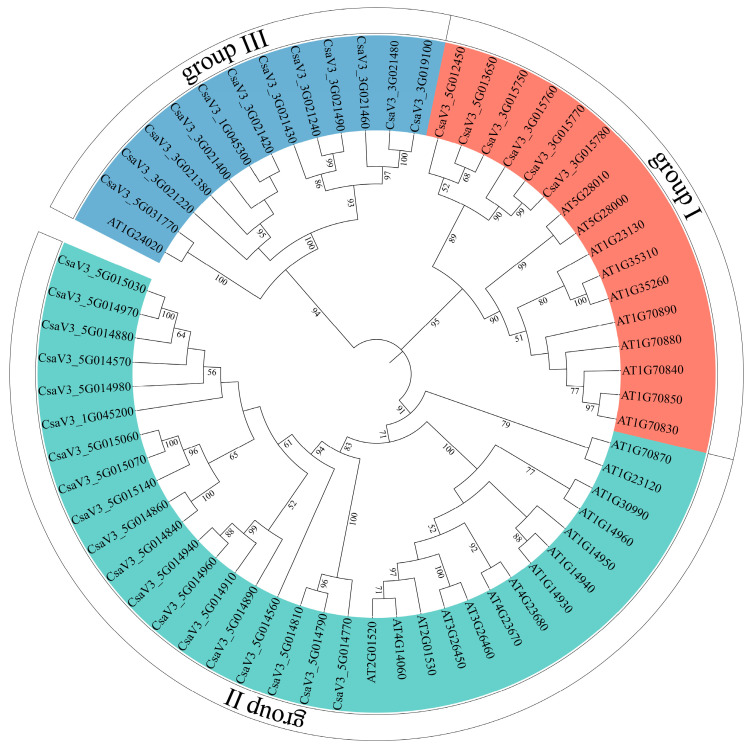
Phylogenetic relationship of MLP proteins from cucumber and *Arabidopsis thaliana*. The number on the branch indicates the bootstrap value.

To further understand their origin and evolution, the phylogenetic relationship between cucumber and *Arabidopsis thaliana* MLP sequences was constructed. The results showed that 37 cucumber MLP and 25 *Arabidopsis thaliana* MLP protein sequences clustered into three groups. A total of six CsMLPs and 10 AtMLPs clustered within group I, and 19 CsMLPs and 14 AtMLPs clustered within group II. Finally, 12 CsMLPs and one AtMLP clustered within group III (Figure 2).

### 2.4. Analysis of Putative Motifs and Regulatory Elements in CsMLP Proteins

**Figure 3:**
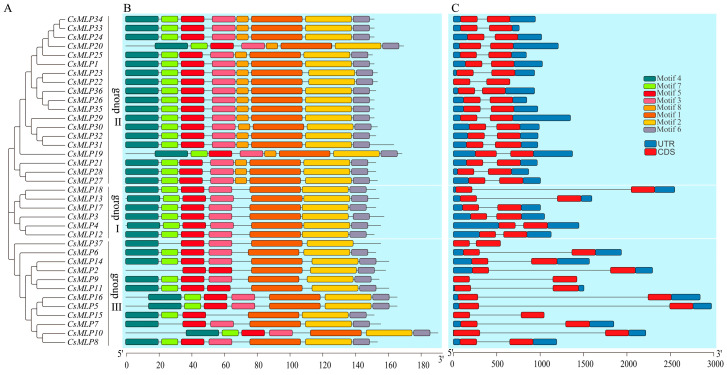
Phylogenetic tree, gene structure, and schematic representation of the conserved motifs in deducing CsMLP proteins. (**A**) the phylogenetic tree of 37 CsMLP proteins. (**B**) the motif compositions of *CsMLP* genes. (**C**) exon/intron architectures of *CsMLP* genes. Exons and introns are represented by red and blue rectangles, respectively. Upstream/downstream regions are indicated by black lines.

MEME motif analysis showed that the protein structures of the CsMLP family were broadly conserved, and eight motifs are widely distributed in all CsMLP proteins. Interestingly, CsMLP members share a similar motif composition within the same phylogenetic group, suggesting that they have similar functions in plants. The proteins of group I contained motifs 1~7, group II contained all eight motifs, and group III contained motifs 1~7; as an exception, two genes (CsMLP37, CsMLP7) missed motif 7, CsMLP15 missed motif 8, and CsMLP2 missed motif 4 and motif 7 (Figure 3 and Figure S3).

**Figure 4:**
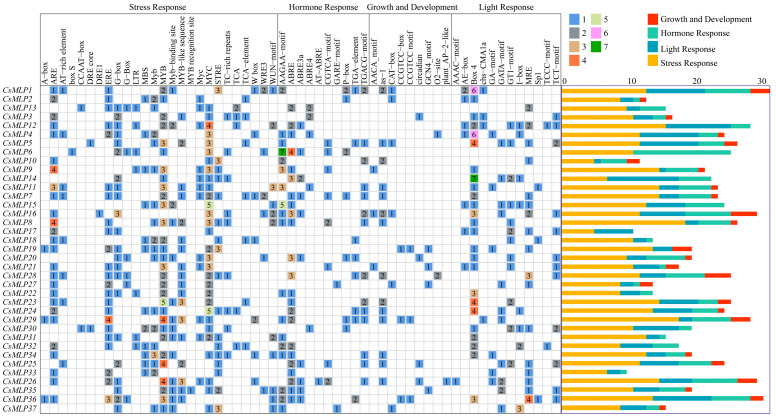
Predicted regulatory elements in the promoter regions of *CsMLP* genes. Promoter sequences (−2000 bp) for 37 *CsMLP* genes are analyzed. The names of the promoters of *CsMLP* genes are shown at the top of the figure. Arabic numerals 1~7 in a square box indicate the number of specific *cis*-elements. The total number of *cis*-elements from different functional categories is marked with a different color.

The promoter regions (2.0 kb) of 37 *CsMLP* genes were searched by TBtools software. The PlantCare-based analysis revealed that all *CsMLP* genes possessed multiple regulatory elements, including stress response elements, hormone response elements, growth and development response elements and light response elements (Figure 4). Among them, the *CsMLP1* had the maximum types of regulatory elements (13 *cis*-elements related to stress response, seven *cis*-elements related to hormone response, three *cis*-elements related to growth and development response, and nine *cis*-elements related to light response), and the *CsMLP33* possessed the minimum types of regulatory elements (seven *cis*-elements related to stress response, one *cis*-element related to hormone response, and two *cis*-elements related to light response).

### 2.5. Transcriptome Analysis and Expression Verification of CsMLP Genes in Response to P. melonis

**Figure 5:**
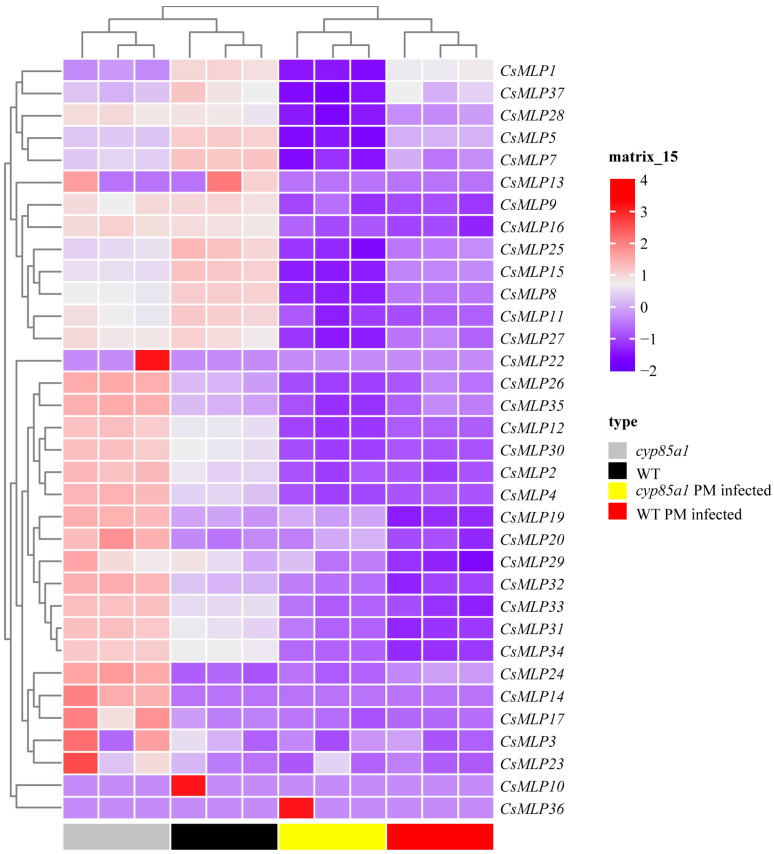
Heat map diagrams of 37 *CsMLP* genes expression. Heatmap colors represent the gene expression (shown as absolute normalized RPKM values).

To explore the role of *CsMLP* genes in response to *P. melonis*, the transcriptome of cucumber hypocotyls inoculated with *P. melonis* at 72 h was used to analyze the expression patterns of the *MLP* genes (Figure 5). Interestingly, most *MLP* genes were significantly down-regulated both in susceptible cucumber genotype *cyp85a1* and resistant wild-type genotype CCMC. Due to the greater inhibitory effect of pathogen infection in *cyp85a1* plants than that in WT plants, the higher expression level of eight *MLP* genes in infected-hypocotyls of WT plants was observed, including *CsMLP1*, *CsMLP5*, *CsMLP7*, *CsMLP15*, *CsMLP8*, *CsMLP25*, *CsMLP28,* and *CsMLP37* (Figure 5). Among eight *MLP* genes, *CsMLP1* and *CsMLP5* showed the greatest differentially expressed levels. *CsMLP1* expression was obviously down-regulated in *cyp85a1* in response to *P. melonis* inoculation but remained stable in WT plants. However, pathogen infection led to great inhibition of *CsMLP5* expression both in WT and *cyp85a1*. qRT-PCR verification showed the same expression patterns (Figure S4).

### 2.6. Protein Sequence Analysis and Subcellular Localization of CsMLP1 and CsMLP5

**Figure 6:**
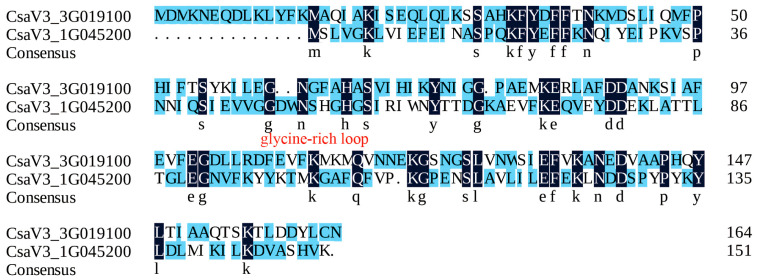
Sequence analysis to CsaV3_1G045200 (CsMLP1) and CsaV3_3G019100 (CsMLP5) in cucumber. The conserved Gly-rich loop of the MLP protein was marked in red. Amino acid residues conserved in two proteins are black shaded.

The length of proteins CsMLP1 and CsMLP5 were 152 and 166 amino acids, respectively. And the predicted molecular mass was 17.32 and 18.94 kDa, and the pI values were 5.27 and 6.01 (Table S1). The sequence alignment analysis revealed a 23.81% similarity between CsMLP1 and CsMLP5. Both CsMLP1 and CsMLP5 contained a glycine-rich loop in the form of a GlyXXXXXGly motif (Figure 6). We constructed a phylogenetic tree of CsMLP1 and CsMLP5 (Figure S5) and members of the MLP family in related species. The result showed that CsMLP1 shares the highest sequence similarity with Arabidopsis MLP1, MLP3 and MLP329, and CsMLP5 shares the highest sequence similarity with Arabidopsis MLP423.

**Figure 7:**
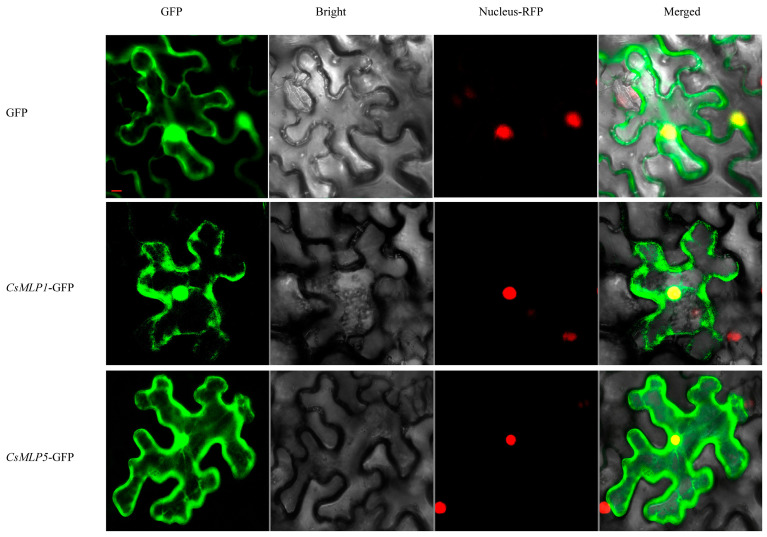
The subcellular localization of CsMLP1 and CsMLP5. Bars, 10 µm.

We investigated the subcellular localizations of the CsMLP1 and CsMLP5 proteins, and confocal imaging revealed that CsMLP1-GFP was found in the cell membrane and nucleus, and CsMLP5-GFP in the cytoplasm and nucleus membrane (Figure 7).

### 2.7. The Effects of Individual Silencing Homologues of CsMLP1 or CsMLP5 on P. melonis Infection in Cucumber

**Figure 8:**
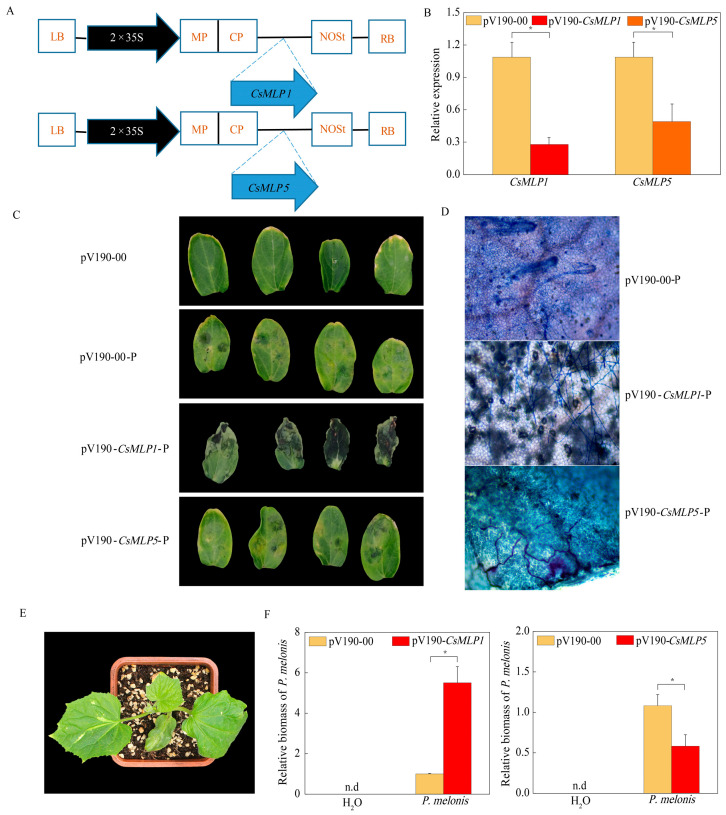
Resistance identification of *CsMLP1*- and *CsMLP5*-silencing cucumber cotyledons on 72 h after *P. melonis* inoculation. (**A**) Schematic representation of the pV190 vector for insertion of *CsMLP1* and *CsMLP5* fragments. (**B**) The transcription levels of *CsMLP1* and *CsMLP5* in the *CsMLP1*-silenced, *CsMLP5*-silenced, and pV190 cotyledons. (**C**) The water-soaking lesion areas were observed in *CsMLP1*-silenced, *CsMLP5*-silenced, and pV190 cotyledons. (**D**) Invasive hyphae extend on the epidermis of *CsMLP1*-silenced, *CsMLP5*-silenced, and pV190 cotyledons stained with lactophenol-trypan blue. (**E**) *CsPDS* gene silencing phenotype in cucumber leaves. The leaf bleaching phenotype was observed 14 days after sprout absorption in pV190-*CsPDS* plants. (**F**) *P. melonis* biomass accumulation in *CsMLP1*-silenced, *CsMLP5*-silenced, and pV190 cotyledons. Data are means ± standard errors from three independent experiments. The asterisks indicate statistically significant differences compared with empty vector control plants (pV190-00) (*p* < 0.05). LB, left border; 2 × 35S, duplicated cauliflower mosaic virus (CaMV) 35S promoter; MP, movement protein; CP, coat protein; NOSt, nopaline synthase terminator; RB, right border.

To confirm whether a loss-of-function of *CsMLP1* and *CsMLP5* affected cucumber resistance to *P. melonis*, we constructed pV190-*CsMLP1*, pV190-*CsMLP5*, and pV190-*CsPDS* vectors to silence endogenous genes in wild-type cucumber CCMC (Figure 8A). Silencing of *PDS* (phytoene desaturase), a key gene in the carotenoid biosynthesis pathway, can result in the photobleaching phenotypes in cucumbers. Therefore, in this study, silenced (pV190-*CsPDS*) cucumber plants were used to monitor the virus-induced gene silencing (VIGS) progression. On 14 days post-agroinfiltration (dpi), the PV190-*CsPDS*-infiltrated plants exhibited photobleached dots (Figure 8E), demonstrating that the *CsPDS* was silenced in the plants. The qRT-PCR results showed that the average gene silencing efficiencies of *CsMLP1* and *CsMLP5* were 72.22% and 51.00% (Figure 8B). Interestingly, silencing of *CsMLP1* and *CsMLP5* expression caused different effects on the resistance of cucumber plants to *P. melonis*. Silencing of *CsMLP1* caused more severe water soaking with necrotic spots compared to that in the cotyledons infected with pV190. In contrast, silencing of *CsMLP5* prevented disease development. The results of the lactophenol-trypan blue stain and *P. melonis* biomass assay showed accumulation level of *P. melonis* in the cotyledons was correlated with the severity of disease symptoms. Extensively branched hyphae were observed on the epidermal cells of *CsMLP1*-silenced cotyledons (Figure 8D), and relative *P. melonis* biomass was 5.49 times that in pV190-inoculated cotyledons (Figure 8F). On the contrary, only a few invasive hyphae were developed in *CsMLP5*-silenced cotyledons, and relative *P. melonis* biomass was 53.74% that in pV190-inoculated cotyledons.

### 2.8. Effects of Transient Overexpression of CsMLP1 or CsMLP5 in Cucumber Cotyledons on P. melonis Infection

**Figure 9:**
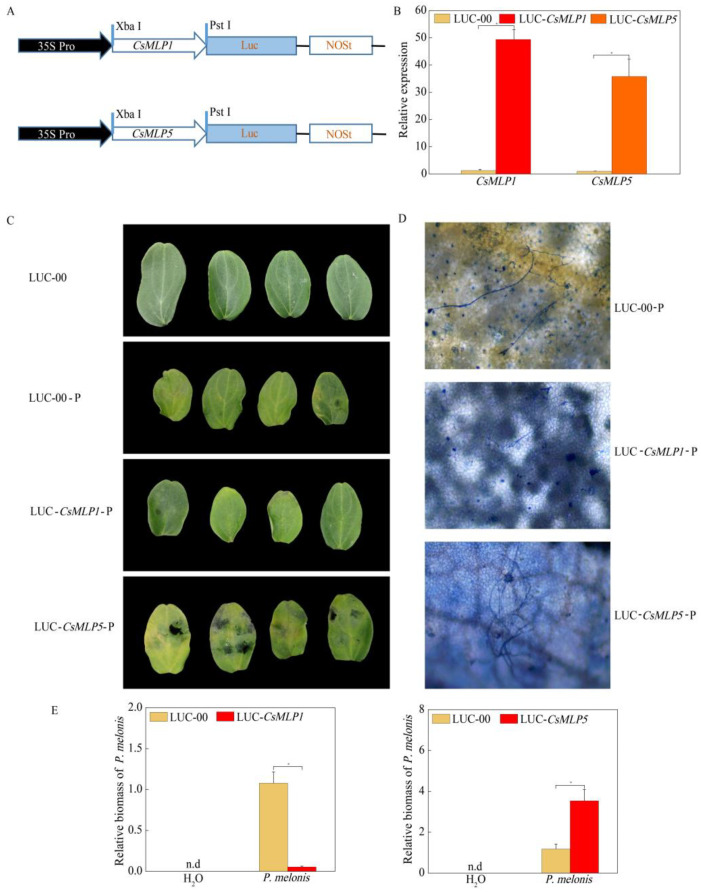
Resistance identification of transient overexpressing plants on 72 h after *P. melonis* inoculation. (**A**) Schematic of the *CsMLP1*-Luc and *CsMLP5*-Luc constructs. *CsMLP1* and *CsMLP5* were between 35S Pro and Luc proteins. (**B**) Transgenic plants were identified by qRT-PCR. (**C**) The water-soaking lesion areas were observed in *CsMLP1*- and *CsMLP5*-transient overexpressing cucumber cotyledons. (**D**) Invasive hyphae extend on the epidermis of *CsMLP1*- and *CsMLP5*-transient overexpressing cucumber cotyledons stained with lactophenol-trypan blue. (**E**) *P. melonis* biomass accumulation in *CsMLP1*- and *CsMLP5*-transient overexpressing cucumber cotyledons. Data are means ± standard errors from three independent experiments. The asterisks indicate statistically significant differences compared with empty vector control plants (LUC-00) (*p* < 0.05). XbaI and Pst I, restriction endonuclease; Luc, luciferase; NOSt, NOS terminator.

To further explore whether the resistance responses were affected by *CsMLP1* and *CsMLP5* in cucumber cotyledons, overexpression vectors containing *CsMLP1* and *CsMLP5* (Figure 9A) fused with luciferase (LUC) were constructed. Agrobacterium carrying LUC-CsMLP1, LUC-CsMLP5, and LUC-00 were infiltrated into the cotyledons of cucumber CCMC. At 48 h, the RT-qPCR results showed that the expression of the CsMLP1/CsMLP5 genes was greatly enhanced compared with that of the LUC-00-injected cucumber cotyledons, indicating *CsMLP1* and *CsMLP5* were effectively transiently overexpressed in cucumber cotyledons (Figure 9B). The defense resistance levels of *CsMLP1*/*CsMLP5*-overexpressing cucumber cotyledons were evaluated in isolated cotyledons inoculated with *P. melonis* for 72 h. *CsMLP5*-overexpressing in CCMC plants aggravated disease symptoms with expanded necrotic spots compared with LUC-00-infiltrated plants (Figure 9C). Extensively branched hyphae were developed in *CsMLP5*-overexpressing cotyledons, and relative *P. melonis* biomass was 3.00 times that in LUC-00-infiltrated cotyledons. On the contrary, *CsMLP1*-overexpressing showed a milder disease symptom. Only a few invasive hyphae were observed in *CsMLP1*-overexpressing cotyledons, and relative *P. melonis* biomass was 4.99% of that in LUC-00-injected cotyledons (Figure 9D,E).

## 3. Discussion

Plant MLPs play a role in the transport of hydrophobic compounds as well as abiotic and biotic stress resistance [6]. In this study, we performed a genome-wide identification of MLP in cucumber. Functional analysis revealed that CsMLPs might play a central role in biotic and abiotic stress response, and *CsMLP1* and *CsMLP5* participated in cucumber plants' defense against *P. melonis* infection.

Since the first MLP proteins were isolated from the latex of opium poppy (*Papaver somniferum*) [22], MLP proteins have been subsequently reported in many dicots, monocots, and conifers [6]. Up to now, MLP has been identified genome-wide in many plant species, such as *Vitis vinifera* [23], *Brassica rapa* [24], *Malus domestica* [25], and *Cucurbita pepo* [21]. In the current paper, 37 cucumber *MLP* genes were identified by homology comparison (Table 1). One gene (CsaV3_5G014830) was removed due to the wrong triplet ATG and too-short nucleotide chains, though it was simply summarized that 38 MLP proteins were found in cucumber [25]. The chromosome locations of the remaining 37 MLPs were mapped unevenly on three cucumber chromosomes. Only two chromosomes were involved in gene duplication. It is worth mentioning that nine tandem duplication events were observed, and no segmental duplication was found, indicating that tandem duplication played a role in the expansion of the *CsMLP* gene family in cucumbers (Figure 1) [26]. Previous studies showed that these tandem genes are probably vital for adaptive evolution to quickly changing environments [27]. In the *Vitis vinifera* genome, analysis of the segmental duplication also revealed no duplication events [23]. But in *Malus domestica*, both tandem and segmental duplication played an important role in the expansion of *MdMLP* genes [25].

MEME motif analysis showed that MLP protein structures were broadly conserved in cucumber. However, in the protein structure of Bet v1, the glycine-rich loop in *Cucumis sativus* was limited to GlyxxxxxGly (Figure 3 and Figure S3). Our results were consistent with previous studies that for PR-10 proteins, the glycine-rich loop preserved conserved sequence GlyxGlyGlyxGly(Thr/Ser), but MLPs exhibited less conservation in the glycine-rich loop [28]. Nevertheless, in *Malus domestica*, a conserved glycine-rich loop (GlyxGlyGlyxGlyThr) was found in MLP proteins [25]. Another divergence from the standard PR-10 profile is the existence of more than one Bet v1 domain in MLP homologs [28]. However, only one Bet v1 domain was found in MLP proteins in *Cucumis sativus* (Figure S2), *Malus domestica* [25] and *Brassica rapa* [24], but up to four Bet v1 domains were found in *Cucurbita pepo* [21]. These results suggest that MLP protein is evolutionarily conserved and most diverse among species.

Accumulated lines of evidence show that MLPs play crucial roles in numerous abiotic stresses containing drought and salt and resistance against pathogens, including infectious fungi, bacteria, viruses, and phytoplasma, by the induction of defense-related genes [6]. Promoter sequences are the key elements in the activation of certain gene expressions. In *Cucurbita pepo*, multiple *cis*-acting regulatory elements of *MLP-PG1* related to pathogen response were found. And the promoter activity of *MLP-PG1* was significantly enhanced in leaves of transgenic tobacco plants infected with *Pseudomonas syringae* pv. *tabaci* [9]. Regulatory element analysis revealed that the promoter regions of *CsMLP* genes were associated with light, hormone, growth and stress responsiveness. It was worth noting that *CsMLPs* harbored several pathogen-induced promoters, including MYB, W box, TC-rich repeats and WUN motif and stress-related plant hormones (Figure 4), which were frequently found in the promoters of pathogen-induced genes in plants [29,30]. These results indicate that *CsMLPs* could widely participate in the cucumber defense response to pathogens.

Host plant resistance is a wanted element in an integrated management program because of its ease of use and lack of environmental impact. Until now, commercially acceptable resistant host varieties to *Phytophthora* spp. were extremely lacking. Mining disease-resistance genes might provide a basis for resistance breeding [1]. The global transcription analysis of resistant WT and hypersensitive *cyp85a1* indicated a strong reprogramming of *MLPs* expression in response to *P. melonis* infection (Figure 5), suggesting a potential role of *CsMLPs* in disease defense response. Furthermore, knockdown of *CsMLP1*, down-regulated only in hypersensitive *cyp85a1* after *P. melonis* infection, abolished the disease tolerance in cucumber cotyledons and transient overexpression of *CsMLP1* enhanced disease tolerance of the transgenic cucumber plants. On the contrary, the silence of *CsMLP5*, down-regulated both in WT and *cyp85a1* after *P. melonis* infection, markedly increased disease resistance, and overexpression of *CsMLP5* arrested the Phytophthora resistance. Interestingly, the expression level of *CsMLP1* (average FPKM value 37.87) was greatly lower than that of *CsMLP5* (average FPKM value 753.92) in non-infected WT plants. So, it seems that constitutive expression of *CsMLP1* and *CsMLP5* is not correlated with *P. melonis* resistance, but the response pattern of these two genes to *P. melonis* inoculation might contribute to disease resistance. Furthermore, the phylogenetic analysis showed that *CsMLP1* is homogeneous to stress-related genes *MLP1, MLP3* and *MLP329* of Arabidopsis (Figure 2 and Figure S4). It was reported that Arabidopsis T-DNA insertion mutants *Atmlp1*-1, *Atmlp3*-2 and double mutants *Atmlp1*-1/*Atmlp3*-2 showed increased infection of *Rhizoctonia solani* [10]. And Arabidopsis endoplasmic reticulum- and nuclear-localized protein MLP329 could function as a positive regulator of primary seed dormancy [31]. *CsMLP5* is homogeneous to *AtMLP423* (Figure S5). T-DNA knockout insertion mutant *mlp423* is critical for normal Arabidopsis development and resulted in mild alterations in leaf curvature of Arabidopsis [32]. *MdMLP22*, the homogeneous gene of *AtMLP423*, was significantly reduced by AAAP fungus infection [25]. Collectively, our results suggested that *CsMLP1* and *CsMLP5* play important roles in response to Phytophthora infection. Moreover, CsMLP1-GFP fusion proteins were observed predominantly in the nucleus and weaker fluorescence in the membrane as well, supporting that CsMLP1 might function as a transcriptional regulator. Nevertheless, the mechanism of the differential role of CsMLP1 and CsMLP5, which were localized to different cellular compartments, needs further studies. It is well-known that MLP proteins can bind various hydrophobic compounds, including steroids [6,33]. Li et al. [17] also reported that foliar applied 24-epibrassinolide upregulated MLP-like protein 328 not only in the leaves treated with 24-epibrassinolide but also in distant leaves and phloem sap in *Cucumis sativus*, suggesting 24-epibrassinolide promoted the transport of MLPs to mediate brassinosteroid-regulated systemic resistance. Our Transcriptome dynamics also showed that *CsMLP1* and *CsMLP5* expression were significantly down-regulated in the healthy hypocotyl of brassinosteroid-deficient mutant *cyp85a1* compared with WT (Figure 5), suggesting a possible interaction of brassinosteroid and *CsMLPs*. In addition, our previous study showed that root-applied 24-epibrassinolide effectively arrested the *P. melonis* invasion in cucumber hypocotyls [34]. Thus, we hypothesize the potential interaction of CsMLP proteins and brassinosteroids in counteracting Phytophthora attack.

## 4. Materials and Methods

### 4.1. Plant Materials and Inoculations

The cucumber WT line ‘CCMC’ (*Cucumis sativus* L.) and the BR-deficient mutant *cyp85a1* (encoding BR-C6-oxidase in the BR biosynthesis pathway) were grown in the growth chamber (model RXZ 500-D with fluorescent lamps, Ningbo, China) under a 16/8 h light/dark photoperiod, 70% relative humidity and 26 °C/20 °C (day/night) as described in our previous study. The *cyp85a1* was EMS mutated CCMC isogenic lines and kindly provided by Dr. Yuhong Li (Northwest A & F University, Yangling, Shanxi, China) [35].

For RNA sequence analysis and qRT-PCR verification, the WT and *cyp85a1* mutant plants were inoculated by adding 20 mL of zoospore suspensions (1 × 10^4^ zoospores per mL of deionized water) to the substrate surface (peat: perlite, 3:1 by volume) near the stem base at 2-leaf stages. The WT was a North China-type cucumber with high disease resistance against *P. melonis*, and mutant *cyp85a1* exhibited high susceptibility (Figure S1). The raw RNA-Seq reads have been deposited in the National Center for Biotechnology Information BioProject database (http://www.ncbi.nlm.nih.gov/bioproject (accessed on 13 July 2022)) with ID PRJNA858502.

For VIGS and transient overexpression verification, the suspension drop method was used for pathogen inoculation. When the cotyledons were 14 days old, the cotyledons were detached from WT line ‘CCMC’ for inoculation. A total of 2 droplets (3 × 10³ zoospores per mL of water, 10 μL per droplet, and 1 droplet per cotyledon lobe) were deposited on every seedling using a micropipette. Zoospore suspensions were prepared as described in our previous study [34].

### 4.2. Bioinformatic Analysis

#### 4.2.1. Identification of the *MLP* Genes in Sequenced Cucumber

To identify *MLP* family genes in cucumber, MLP protein sequences from all reviewed plants on the UniProt database (https://www.uniprot.org (accessed on 2 November 2022)) were used to search against the cucumber genomic database (http://cucurbitgenomics.org/ (accessed on 2 November 2022)) using TBtools software (version 1.0987663, South China Agricultural University, Guangzhou, China) (E-value < e−5). All candidate MLP sequences were used to search against NCBI’s SwissPort database (https://blast.ncbi.nlm.nih.gov/Blast.cgi?PROGRAM=blastp (accessed on 2 November 2022)) and further confirmed by the databases, including the Pfam protein family, SMART, and UniProt.

#### 4.2.2. Analysis of Physicochemical Properties of Proteins

The physical location of the MLP chromosomes, as well as the number of exons, were extracted from the cucumber genome annotation file using TBtools software (version 1.0987663), and protein lengths were then calculated. Isoelectric point (pIs) and molecular weight (MWs) were all examined using the ExPASy-ProtParam website (https://web.expasy.org/protparam/ (accessed on 2 November 2022)) [36].

#### 4.2.3. Phylogenetic Analysis

The amino acid sequences of Arabidopsis MLP proteins were collected from the Arabidopsis Information Resource (https://www.arabidopsis.org, accessed on 14 December 2021). Alignment of all MLP protein sequences was performed with the ClustalW tool in the MEGA X software (version 10.2.6, The Pennsylvania State University, University Park, PA, USA). Based on the neighbor-joining method with a 1000 bootstrap value and pairwise deletion, the phylogenetic tree was constructed by the MEGA X software (version 10.2.6) [37]. Then, visualization of the phylogenetic tree was carried out using the Interactive Tree of Life software (iTOL v6) (https://itol.embl.de/ (accessed on 2 November 2022)) [38]. Based on the evolutionary tree, we classified the MLPs of cucumber. The evolutionary trees of CsMLP1 and CsMLP5 were separately constructed and visualized in the same way as above, and their sequences were aligned and visualized using DNAMAN software (version 6.0.3.99).

#### 4.2.4. Gene Structure Analysis and Conserved Motif Prediction

Gene structure comparison was done via the Gene Structure Display Server [39]. For motif identification, the complete amino acid sequences of CsMLPs were analyzed using the MEME Suite (https://meme-suite.org/meme/meme_5.4.1/tools/meme, accessed on 22 June 2022) [40]. The number of motifs was set to 8, and the other parameters were set as default values. TBtools software (version 1.0987663) was used to show gene structure and conserved motifs [41].

#### 4.2.5. *Cis*-Acting Element Analysis

The upstream regions (2.0 kb) of the *CsMLP* genes were extracted from the cucumber genome database. The regulatory element components were predicted using the PlantCare online software (https://bioinformatics.psb.ugent.be/webtools/plantcare/html/, accessed on 20 May 2022) [42]. Visualization of *cis*-acting elements was performed using the ggplot2 package in RStudio.

#### 4.2.6. Collinearity and Gene Duplication Analysis of the MLPs

BLASTP and MCScanX software were then used to examine the intra-species collinearity relationship of cucumbers. The synonymous and non-synonymous substitution (Ka/Ks) of the collinear gene pairs were determined using the ParaAT (2.0) and KaKs_Calculator software (Version 3.0, 2021) [43,44].

### 4.3. RNA-Seq Analysis

By analyzing our transcriptome data, we determined the expression patterns of the members of the *CsMLPs* family in response to *P. melonis* infection in WT and mutant *cyp85a1* hypocotyls. TBtools software (version 1.0987663) was used to visualize the results.

### 4.4. Subcellular Localization of CsMLP1 and CsMLP5

The full-length ORF of the CsMLP1 and CsMLP5 without a stop codon was separately PCR amplified using primers (Table S1) and then inserted into vector pCAMBIA1300-GFP to generate a fusion construct. After the identification of the correct sequence, it was transformed into *Agrobacterium tumefaciens* EHA105 by electroporation. EHA105 cells containing pCAMBIA1300-GFP (control vector), pCAMBIA1300-CsMLP1-GFP, and pCAMBIA1300-CsMLP5-GFP were inoculated with 30-day-old *N. benthamiana* leaves. Co-localization of recombinant vector and Nucleus-RFP to evaluate nuclear localization. Tobacco was cultured for 48 h and then observed with confocal microscopy (LSM 800, Zeiss, Jena, Germany) [45].

### 4.5. VIGS Vectors Construct and Agrobacterium-Mediated Virus Infection

Two targeted *MLP* genes, *CsMLP1* and *CsMLP5,* were silenced using pV190 VIGS vectors to analyze their functions during *P. melonis* infection in cucumber plants [46]. The VIGS vector was generously provided by Dr. Qinsheng Gu of Zhengzhou Fruit Research Institute, Chinese Academy of Agricultural Sciences (Zhengzhou, China). Briefly, approximately 300 bp fragments of these genes were PCR amplification using the gene-specific primers (Table S1). The resulting PCR products were ligated into the pV190 vector using the ClonExpress II One Step Cloning Kit (Vazyme, C112, Nanjing, China). The constructed plasmids were transformed into *Agrobacterium tumefaciens* strain GV3101 (TOLOBIO, CC96304, Shanghai, China). pV190-*CsPDS*, where *CsPDS* (XM_011654729) encoded phytoene desaturase, was used as a positive control to monitor the silencing efficiency based on the photo-bleaching phenotypes. For VIGS, the sprout absorption method was performed as previously described by Liao [47]. Plants infiltrated with an *Agrobacterium* culture carrying the empty pV190 VIGS vector were used as controls.

At the 2-leaf stage, the pV190-*CsPDS*-infiltrated plants showed highly uniform bleaching in newly emerged leaves. At the same time, the cotyledons were sampled to detect the expression of *CsMLP5* and *CsMLP1* to verify the silencing effect of these vectors. The cotyledons that showed less than 50% transcript levels of control plants were detached and used for *P. melonis* infection. The mock-inoculation of cotyledons using sterile water instead of zoospore suspension was also performed as a control.

### 4.6. CsMLP-Luciferase (LUC) Fusion Overexpression Vector Construct

The pCAMBIA 3301 vector with luciferase (LUC) was used to construct LUC-CsMLP5 and LUC-CsMLP1 to transiently expression target genes under the control of the CaMV 35S promoter in the cotyledons of cucumber plants. The gene-specific primers are listed in Table S1. The constructed plasmids were introduced into the *Agrobacterium tumefaciens strain* GV3101 (TOLOBIO, CC96304). Agrobacterium-meditated transformation with the LUC-00, LUC-CsMLP5 and LUC-CsMLP1 was carried out as described by Yu et al. [48].

### 4.7. In Vivo Analyses of P. melonis Infection

For the visualization of fungal structures, inoculated cotyledons epidermis was stained with lactophenol-trypan blue as Tao’s described [49] at 72 h after inoculation with pathogens.

### 4.8. DNA Extraction and Fungal Biomass Quantification

DNA extraction and detection of *P. melonis* from cucumber cotyledons were performed as previously Wang’s described [50]. Briefly, pathogen biomass was accurately quantified by amplifying the internal transcribed spacer of nuclear ribosomal DNA of *P. melonis* (Table S1).

## 5. Conclusions

We have identified 37 *MLP* genes in the cucumber genome and then comprehensively elucidated their gene structure, phylogenetic, conserved motif prediction and *cis*-elements prediction. Additionally, transcriptome profile analysis revealed a wide involvement of *MLPs* in response to *P. melonis* infection. Importantly, the overexpression of *CsMLP1* increases resistance to *P. melonis* infection, whereas *CsMLP5* weakens this defense response. Together, these findings lay a solid foundation for the functional characterization of *CsMLP* genes in cucumber, especially in response to *P. melonis*. Although additional research is required, for instance, the stable overexpression and knockout of cucumber plants are created to elucidate the functions of MLPs, and the role of MLPs in brassinosteroid-induced blight resistance, the insight gained in this work can deepen our understanding of MLPs ultimately guide effective cultivation and precision breeding.

## Figures and Tables

**Table 1:** Characteristics of MLP family members in cucumber.

Gene Name	Gene ID	Genomic Location	Group	Exon	Size (aa)	MW (Da)	pI
*CsMLP1*	CsaV3_1G045200	chr1:3088298030884006	II	2	152	17,321.7	5.27
*CsMLP2*	CsaV3_1G045300	chr1:3104944331051738	III	2	159	18,130.5	4.92
*CsMLP3*	CsaV3_3G015760	chr3:1170652211707573	I	2	158	18,095.7	7.02
*CsMLP4*	CsaV3_3G015780	chr3:1171361611715063	I	2	156	17,851	5.06
*CsMLP5*	CsaV3_3G019100	chr3:1478970014792674	III	2	166	18,937.5	6.01
*CsMLP6*	CsaV3_3G021220	chr3:1796757617969512	III	2	153	17,031	4.53
*CsMLP7*	CsaV3_3G021430	chr3:1841356518415414	III	2	156	17,568.1	5.64
*CsMLP8*	CsaV3_3G021490	chr3:1850282518504016	III	2	154	17,551.9	6.5
*CsMLP9*	CsaV3_3G021380	chr3:1832351218324936	III	2	155	17,625.2	9.21
*CsMLP10*	CsaV3_3G021240	chr3:1800448218006699	III	2	191	21,748.9	7.29
*CsMLP11*	CsaV3_3G021420	chr3:1838331318384818	III	2	161	18,271.8	6.81
*CsMLP12*	CsaV3_3G015770	chr3:1171026011711387	I	2	152	17,527	6.79
*CsMLP13*	CsaV3_3G015750	chr3:1170241111704007	I	2	155	17,753	5.71
*CsMLP14*	CsaV3_3G021400	chr3:18350283 18351850	III	2	161	18,201.7	7.56
*CsMLP15*	CsaV3_3G021460	chr3:18452834 18453880	III	2	152	17,164.5	5.65
*CsMLP16*	CsaV3_3G021480	chr3:18490521 18493364	III	2	166	18,842.3	5.93
*CsMLP17*	CsaV3_5G012450	chr5:7954071 7955075	I	2	153	17,234.8	6.74
*CsMLP18*	CsaV3_5G013650	chr5:10398882 10401432	I	2	153	17,317.6	6.68
*CsMLP19*	CsaV3_5G014560	chr5:11564949 11566322	II	2	169	19,361.1	7.71
*CsMLP20*	CsaV3_5G014570	chr5:11591960 11593169	II	2	170	19,657.3	5.55
*CsMLP21*	CsaV3_5G014770	chr5:11895386 11896353	II	2	153	17,842.2	5.53
*CsMLP22*	CsaV3_5G014840	chr5:11949639 11950291	II	2	154	17,541	6.63
*CsMLP23*	CsaV3_5G014860	chr5:11957037 11957973	II	2	154	17,644.2	6.88
*CsMLP24*	CsaV3_5G014880	chr5:11995291 11996308	II	2	152	17,336.5	4.87
*CsMLP25*	CsaV3_5G014980	chr5:12151706 12152547	II	2	151	17,473.8	5.97
*CsMLP26*	CsaV3_5G015060	chr5:12245033 12245879	II	2	152	17,545.9	6.01
*CsMLP27*	CsaV3_5G014810	chr5:11931904 11932908	II	2	154	17,791.2	6.23
*CsMLP28*	CsaV3_5G014790	chr5:11918471 11919339	II	2	153	17,616.9	5.86
*CsMLP29*	CsaV3_5G014890	chr5:12018406 12019756	II	2	152	17,653.3	7.04
*CsMLP30*	CsaV3_5G014910	chr5:12033997 12034987	II	2	154	17,729.4	8.1
*CsMLP31*	CsaV3_5G014940	chr5:12076782 12077755	II	2	164	18,628.3	6.97
*CsMLP32*	CsaV3_5G014960	chr5:12114037 12115013	II	2	153	17,502.9	8.75
*CsMLP33*	CsaV3_5G015030	chr5:12203912 12204671	II	2	152	17,362.5	4.89
*CsMLP34*	CsaV3_5G014970	chr5:12146702 12147647	II	2	152	17,376.5	4.89
*CsMLP35*	CsaV3_5G015070	chr5:12261080 12262053	II	2	152	17,561.9	6.01
*CsMLP36*	CsaV3_5G015140	chr5:12327275 12328211	II	2	153	17,774.2	5.58
*CsMLP37*	CsaV3_5G031770	chr5:25888965 25889508	III	2	156	17,542.7	4.73

## Data Availability

The raw RNA-Seq reads have been deposited in the National Center for Biotechnology Information BioProject database (http://www.ncbi.nlm.nih.gov/bioproject (accessed on 13 July 2022)) with ID PRJNA858502.

## Supplementary Materials

The following supporting information can be downloaded at: https://www.mdpi.com/article/10.3390/ijms24010784/s1.
