# Supplementary material for: Comprehensive Analysis of Major Latex-Like Protein Family Genes in Cucumber (Cucumis sativus L.) and Their Potential Roles in Phytophthora Blight Resistance
Source: Int J Mol Sci. 2023 Jan 2;24(1):784. doi: 10.3390/ijms24010784 (PMC9821209; doi:10.3390/ijms24010784)
Supplement: Supplementary file 1 [file ijms-24-00784-s001.zip › ijms-2107713-supplementary.pdf]

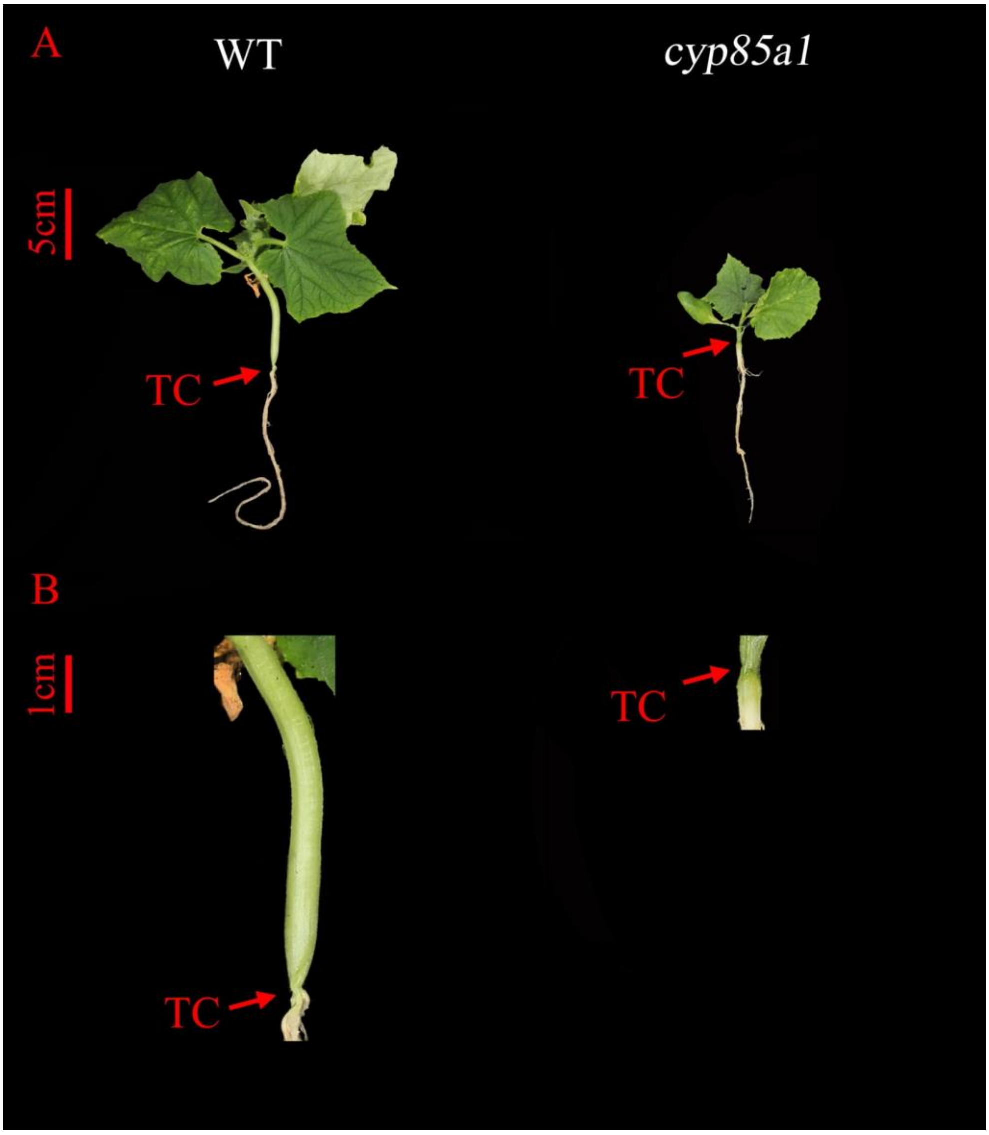

Figure S1. Phenotypes of *P. melonis* infected mutant *cyp85a1* and WT plants 72 h after inoculation. Abbreviations: TC, tissue collapse.

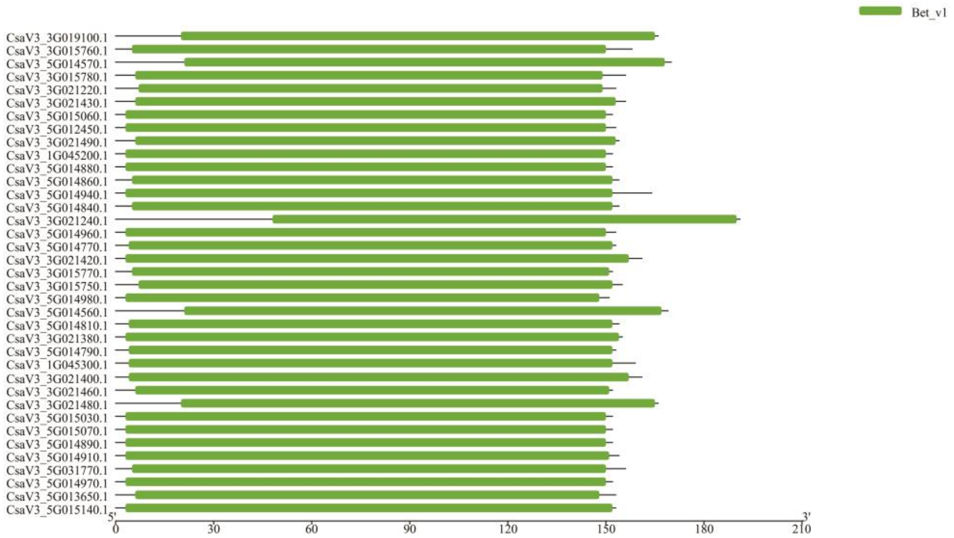

Figure S2. Analysis of CsMLP domain. All 37 CsMLP proteins contain one Bet v1 domain.

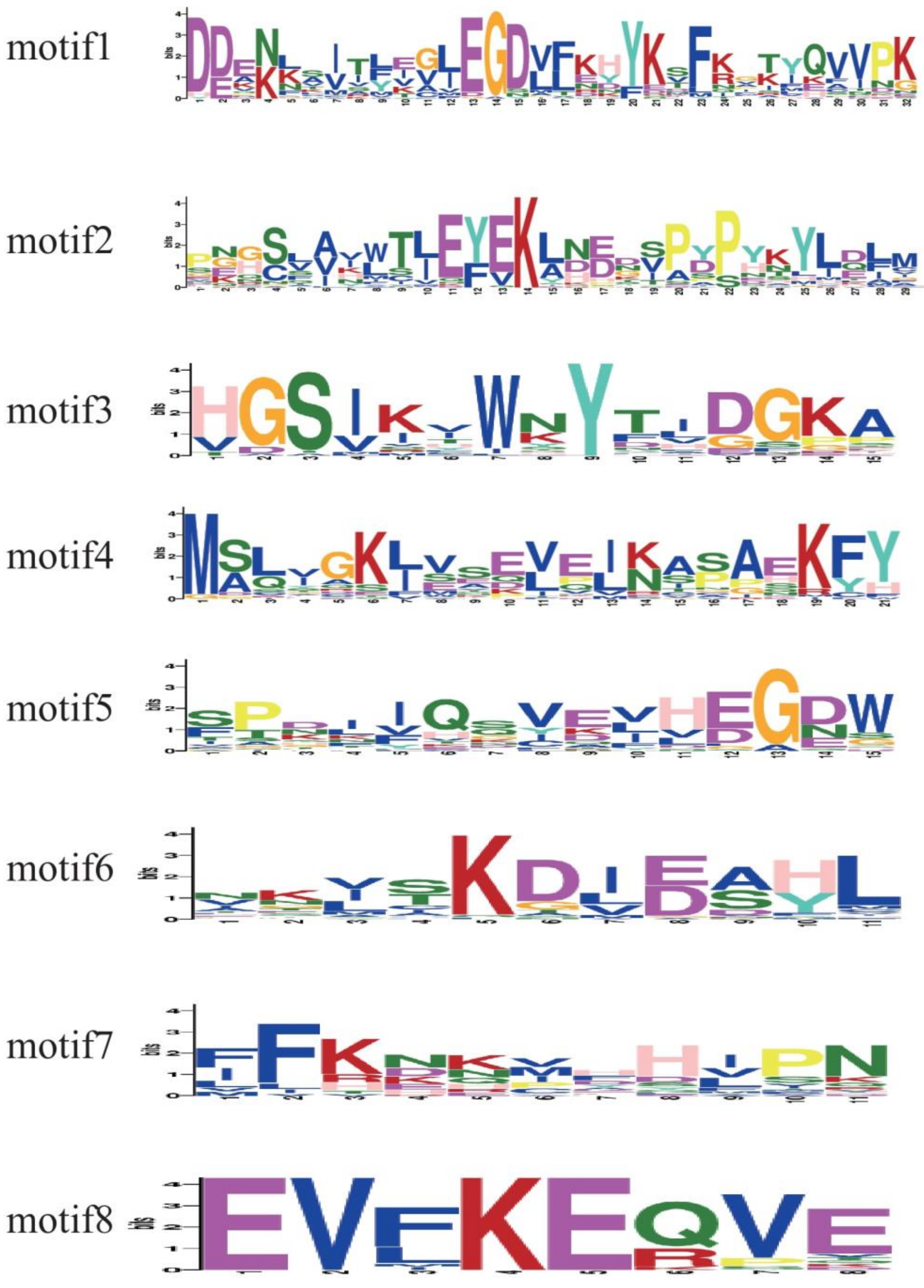

Figure S3. The pattern identification of eight conservative sequences.

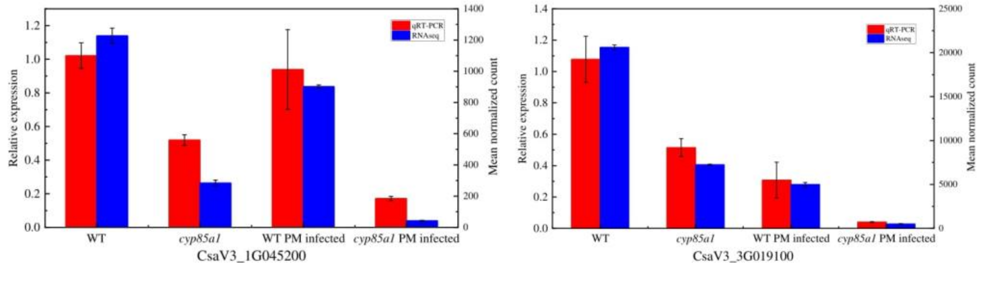

Figure S4. Validation of RNA-seq gene expression of *CsMLP1* and *CsMLP5* using quantitative real-time PCR (qRT-PCR).

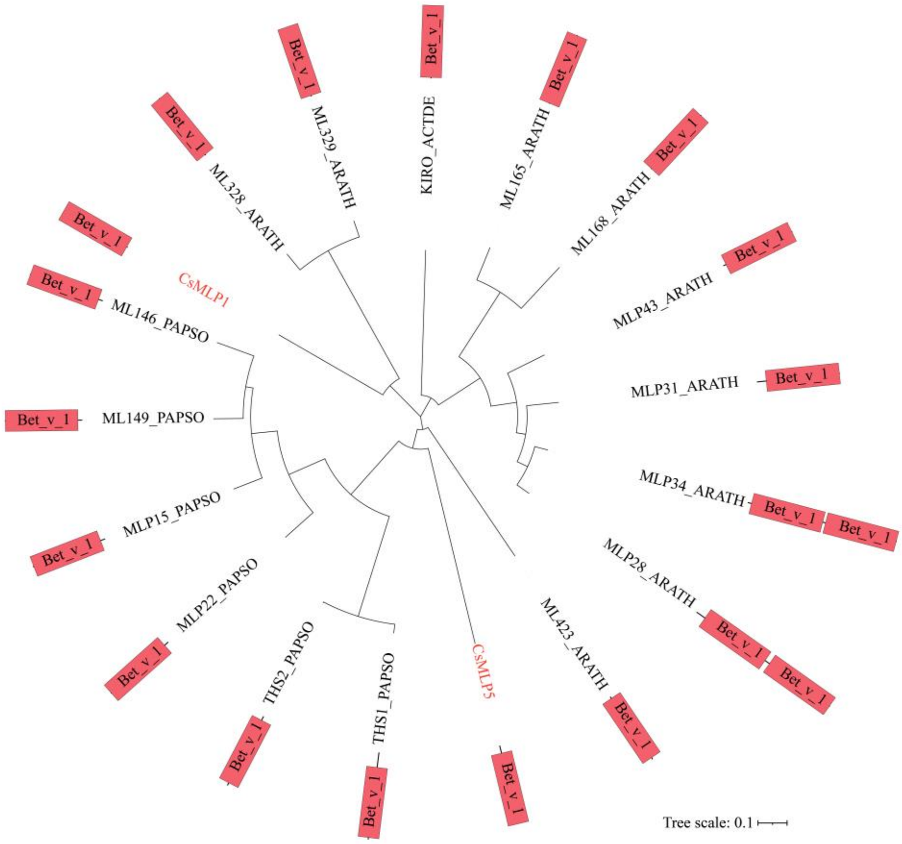

Figure S5. Alignment of CsMLP1 (CsaV3_1G045200) and CsMLP5 (CsaV3_3G019100) to MLP protein sequences from all reviewed plants on UniProtKB/Swiss-Prot database. The conserved motifs are represented by rectangles.

Table S1. List of primers used in this study.

Analysis	Primer Name	Sequence (5′-3′)
Sequencing of the cDNA of CsMLP5	CsMLP5-F	ATGGACATGAAGAATGAGCAAG
	CsMLP5-R	GTTGTTGCAAAGGTAATCATCAAG
Sequencing of the cDNA of CsMLP1	CsMLP1-F	ATGTCTCTAGTTGGGAAACTTG
	CsMLP1-R	TTTAACATGCGATGCAACGT
qRT-PCR for CsMLP5	RT-CsMLP5-F	TGGACATGAAGAATGAGCAAGATTTGA
	RT-CsMLP5-R	TGAAAAAGTCGTAGAATTTGTGGGCAG
qRT-PCR for CsMLP1	RT-CsMLP1-F	GTTGTTGGAGGAGATTGGAACAGCC
	RT-CsMLP1-R	TCCAATCCAGTTAGAGTGGTTGCCA
pCAMBIA1300 vector for CsMLP1	pCAMBIA1300-GFP-CsMLP1-F	gagaacacgggggacaagcttATGTCTCTAGTTGGGAAACT
	pCAMBIA1300-GFP-CsMLP1-R	accggtttctctagaggtaccTTTAACATGCGATGCAACGT
pCAMBIA1300 vector for CsMLP5	pCAMBIA1300-GFP-CsMLP1-F	gagaacacgggggacaagcttATGGACATGAAGAATGAGCA
	pCAMBIA1300-GFP-CsMLP1-R	accggtttctctagaggtaccGTTGTTGCAAAGGTAATCATC
CsMLP5-silencing vector	pV190-CsMLP5-F	aggactttacttaatggatccAGGACTTTACTTAATGGATCC

	pV190-CsMLP5-R	ctagacctataactggatccCCTAGACCTATAACTGGATCC
CsMLP1-silencing vector	pV190-CsMLP1-F	aggactttacttaatggatccAGGACTTTACTTAATGGATCC
	pV190-CsMLP1-R	ctagacctataactggatccCCTAGACCTATAACTGGATCC
CsMLP5-overexpression vector	Luc-CsMLP5-F	ggggactgctctagaggatccATGGACATGAAGAATGAGCAAG
	Luc-CsMLP5-R	tttggcgtcttccataagcttGTTGTTGCAAAGGTAATCATCAAG
CsMLP1-overexpression vector	Luc-CsMLP1-F	ggggactgctctagaggatccATGTCTCTAGTTGGGAAACTTG
	Luc-CsMLP1-R	tttggcgtcttccataagcttTTTAACATGCGATGCAACGT
Fungal biomass quantification	Pm1/Pm2-F	ACTGGATCATGAGCCCACC
	Pm1/Pm2-R	GGTTCACCAGCCCATACCA
	Csa-UBI-F	CACCAAGCCCAAGAAGATC
	Csa-UBI-R	TAAACCTAATCACCACCAGC

Lower cases in the sequences, ARMS primer sequence of the construct.
